# Spécificité de la transformation sarcomateuse de la maladie de Recklinghaussen: a propos de deux cas et revue de la littérature

**DOI:** 10.11604/pamj.2013.15.73.2593

**Published:** 2013-06-25

**Authors:** Mouna Bourhafour, Imane Bourhafour, Meryam Ben Ameur El Youbi, Hind M'Rabti, Nourredine Benjaafar, Hassan Errihani

**Affiliations:** 1 Service d'oncologie médicale, Institut national d'oncologie, Rabat-Maroc; 2 Service de radiothérapie, Institut national d'oncologie, Rabat-Maroc

**Keywords:** Maladie de Recklinghausen, transformation sarcomateuse, neurofibromatose de type 1, Recklinghausen disease, sarcomatous transformation, neurofibromatosis type 1

## Abstract

5 à 10% des patients atteints de neurofibromatose de type 1 (NF1) développent des tumeurs malignes des gaines des nerfs périphériques (Malignant peripheral nerve sheath tumor: MPNST) contre 0.001% dans la population générale. A travers deux observations et une revue de la littérature nous discuterons la spécificité de la transformation sarcomateuse au cours de la neurofibromatose de type 1.

## Introduction

Les sarcomes des tissus mous (STM) sont des tumeurs rares, caractérisées par une grande hétérogénéité anatomique, histologique et pronostique. Il existe très peu de données sur leur épidémiologie descriptive et analytique. La prédisposition génétique participe à 3% des STM et correspond essentiellement à la neurofibromatose (NF). Nous rapportons les observations de deux patients qui ont été suivis pour sarcomes de tissus mous compliquant la maladie de Recklinghaussen à l'Institut National d'Oncologie de Rabat.

## Patient et observation

### Observation 1

**Figure 1:**
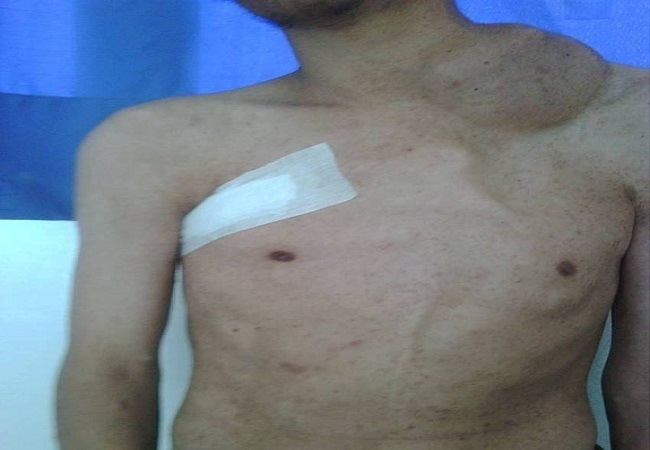
Masse latéro-cervicale gauche mesurant environ 10cm, ferme, douloureuse à la palpation (neurofibrome plexiforme)

**Figure 2:**
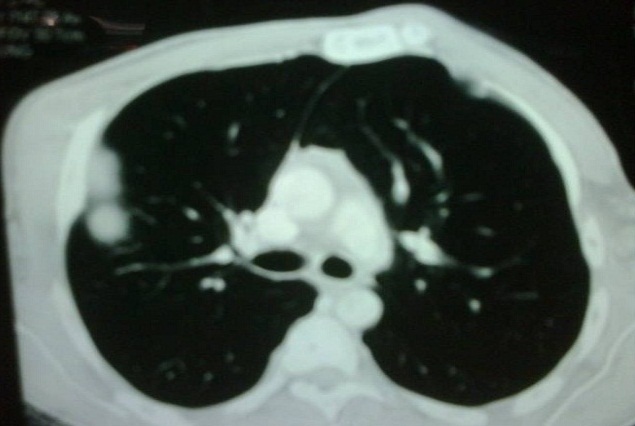
Formation ovalaire, de densité hétérogène, multiloculé, 85 x 84 mm, au niveau da la région sous-claviculaire et axillaire droite et présentant un développement endothoracique

**Figure 3:**
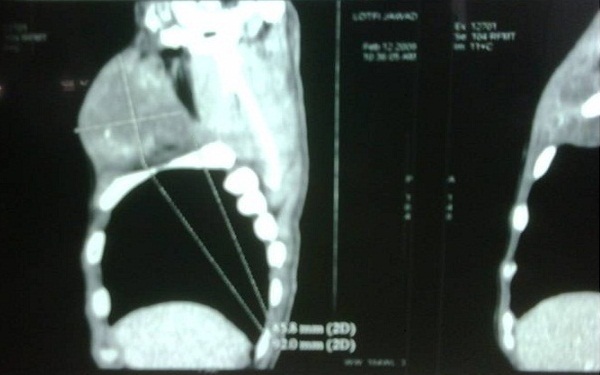
Masse basi-cervicale gauche sus-claviculaire, refoulant les vaisseaux carotidiens et jugulaires en dedans, arrivant jusqu'au niveau de l'apex pulmonair

Monsieur L. J âgé de 26 ans, atteint d'une maladie de Von Recklinghausen depuis son enfance, suivi à l'Institut National d'Oncologie de Rabat pour un Synovialosarcome de la paroi thoracique. Motif de consultation: Douleur et augmentation de la taille d'un neurofibrome latéro-thoracique droit. Signes généraux: Altération de l’état général et amaigrissement non chiffré. A l'examen clinique: Tâches café au lait, neurofibromes cutanés, masses latéro-thoracique doite et latéro-cervicale gauche (Figure 1). TDM thoraco-abdomino-pelvienne: Formation au niveau da la région sous-claviculaire et axillaire droite et une masse basi-cervicale gauche sus-claviculaire (Figure 2, Figure 3). Exérèse de la tumeur de la paroi thoracique + résection de la 3^ème^ et 4^ème^ côte (Chirurgie R2): Synovialosarcome biphasique Grade 3 selon la FNLCC. Biopsie d'un nodule pleuro-pulmonaire: métastase pulmonaire d'un synovialosarcome. Une chimiothérapie de type AI (Adriamycine, Ifosfamide) a été indiquée. Le patient est décèdé avant tout traitement.

### Observation 2

B. M âgé de 35 ans, suivi pour neurofibromatose type I depuis son enfance, suivi à l'INO pour un liposarcome rétropéritonéal. Motif de consultation: Tuméfaction au niveau du flanc gauche augmentant progressivement de volume sans autres signes associés. Examen clinique: Tâches café au lait, neurofibromes cutanés, tuméfaction mollasse du flanc gauche, mesurant environ 15cm de grand axe, indolore, mal limitée, sans signes inflammatoires en regard. TDM thoraco-abdomino-pelvienne: volumineuse formation tumorale solide rétro-péritonéal gauche de 13cm/10cm avec lyse osseuse vertébro-iliaque et envahissement des parties molles. Biopsie de masse paralombaire gauche: Liposarcome myxoïde (MDM2 et PS 100 positive). Une chimiothérapie néo-adjuvante de type AI (Adriamycine, Ifosfamide) a été démarrée.

## Discussion

L’épidémiologie des sarcomes des tissus mous (STM) est complexe et les facteurs de risque sont mal connus. On estime que 1% des STM sont liés à une pathologie génétique [1]. Les génopathies en cause sont diverses: syndrome de Li-Fraumeni, neurofibromatose de type 1 (NF1), rétinoblastome bilatéral, syndrome de Werner (progeria de l'adulte). Il existe très peu de données sur ces associations, sauf pour le rétinoblastome bilatéral et la NF1. L'association la plus courante concerne la maladie de Recklinghausen [2]. La neurofibromatose 1 ou maladie de Recklinghausen (NF1, MIM 162200) est connue depuis plus d'un siècle grâce à l'observation princeps de Von Recklinghausen (1882) qui lui donna son nom. C'est une maladie génétique fréquente qui touche de 1/4000 à 1/3000 individus avec une répartition mondiale homogène et une incidence estimée à 1/2500 naissances [3].

Il s'agit d'une pathologie autosomique dominante, de pénétrance complète, mais d'expressivité variable. La moitié des cas est familiale, l'autre sporadique. Le gène NF1, responsable de la maladie, est localisé en 17q11.2, il code une protéine cytoplasmique: la neurofibromine [4].

Le diagnostic de NF1, chez l'adulte, est en règle facile sur les données de l'examen clinique. La conférence de consensus du National Institute of Health de Bethesda (États-Unis) a précisé sept critères cardinaux dont deux sont nécessaires pour poser le diagnostic: [5]

1. Un apparenté du premier degré atteint (parent, fratrie ou enfant)
2. Au moins 6 tâches café au lait (TCLs) >; 1.5 cm après la puberté >; 0.5 cm avant la puberté
3. Lentigines axillaires ou inguinales
4. Ou Au moins deux neurofibromes quel que soit le type Au moins un neurofibrome plexiforme
5. Gliome du nerf optique
6. Au moins 2 nodules de Lisch (hamartome irien)
7. Une lésion osseuse caractéristique Pseudarthrose, Dysplasie du sphénoïde, Amincissement du cortex des os longs

Les patients atteints de NF 1 ont quatre fois de risque de développer une pathologie tumorale [1]. Il s'agit surtout de transformation en mélanome, phéochromocytome, cancer médullaire de la thyroïde, astrocytome, gliome des voies optiques, tumeur de Wilm, leucémie. La transformation sarcomateuse est exceptionnelle [6]. Les cancers représentent la principale cause de décès prématuré des patients atteints de NF1. La majorité des tumeurs sont bénignes (neurofibromes) mais si les complications tumorales malignes sont rares, ce sont elles qui font la gravité de la NF1 [3]. La répartition des tumeurs malignes est très différente de celle des tumeurs de la population générale: 50% des tumeurs correspondent à des tumeurs du système nerveux central (épendymome, astrocytome, médulloblastome, méningiome, gliome), dont un tiers sont des gliomes des voies optiques. Le risque de neurofibrosarcome est significativement augmenté chez les patients NF1 et il existe également un risque accru de leucémie et de cancers multiples [7].

La prédisposition génétique participe à 3% des STMA et correspond essentiellement à la NF1, qui intéresse essentiellement des tumeurs malignes des gaines nerveuses (anciennement neurofibrome). Ce chiffre de 3% est certainement sous-estimé car il repose sur la mise en évidence d'un phénotype cliniquement évident. Ces TMNP peuvent être multiples. Pour Guccion et al. [8], les TMNP liés à la NF1 présentent une prédominance masculine nette: 8 hommes pour 2 femmes. L’âge au diagnostic est nettement inférieur à l’âge moyen de diagnostic d'un STMA dans la population générale: âge moyen de 29 ans dans la série de Ducatman et al. [9], de 32 ans dans la série de Guccion et al. [8], de 36 ans dans la série de Hruban et al. [10]. Le diagnostic doit être précoce. Il se développe à partir de neurofibromes nodulaires isolés ou plexiformes, cutanés ou viscéraux. Les signes d'appel sont une augmentation rapide de taille d'un neurofibrome ancien ou nouvellement apparu, des douleurs, l'apparition ou la modification de signes neurologiques préexistants. L'excision'biopsie des nodules suspects doit alors être réalisée sans délai.

Le pronostic des TMNP ne semble pas influencé par la présence d'une NF1, avec une survie globale à 5 ans de l'ordre de 40% dans différentes séries [7–11].

Le traitement des tumeurs malignes doit donc être très spécifique et nécessite une prise en charge spécialisée compte tenu du risque théorique d'apparition d'une seconde néoplasie (ostéosarcome, leucémie myéloïde) après un traitement génotoxique (radiothérapie, chimiothérapie).

## Conclusion

Il apparaît que l’épidémiologie analytique des STMA est complexe car les différents sous-types semblent être liés à des facteurs de risque différents. Alors que les progrès diagnostiques et thérapeutiques s'accumulent dans la prise en charge des STMA, les données épidémiologiques, notamment analytiques, restent pauvres.
